# Over-prescription of short-acting β_2_-agonists remains a serious health concern in Kenya: results from the SABINA III study

**DOI:** 10.1186/s12875-023-02030-8

**Published:** 2023-07-08

**Authors:** Jeremiah Chakaya, Jared Mecha, Maarten Beekman

**Affiliations:** 1 Department of Medicine, Therapeutics, Dermatology and Psychiatry, Kenyatta University, Nairobi, Kenya; 2 Department of Clinical Sciences, Liverpool School of Tropical Medicine, Liverpool, UK; 3 University of Nairobi, Nairobi, Kenya; 4 AstraZeneca, The Hague, The Netherlands

**Keywords:** Asthma management, Over-the-counter, Prescription, SABA, Short-acting β_2_-agonist

## Abstract

**Background:**

Despite a high asthma burden in Kenya, insights into asthma management practices, including prescription of short-acting β_2_-agonists (SABAs), are lacking. Therefore, this study describes patient demographics, disease characteristics, and asthma treatment patterns in the Kenyan cohort of the SABA use IN Asthma (SABINA) III study.

**Methods:**

Patients with asthma (aged ≥ 12 years) with medical records containing data for ≥ 12 months prior to the study visit from 19 sites across Kenya were included in this cross-sectional study and classified by investigator-defined asthma severity (guided by the 2017 Global Initiative for Asthma [GINA] recommendations) and practice type (primary/specialist care). Data on severe exacerbation history, prescribed asthma treatments, and over-the-counter (OTC) SABA purchases in the 12 months before the study visit and asthma symptom control at the time of the study visit were collated using electronic case report forms. All analyses were descriptive in nature.

**Results:**

Overall, 405 patients were analyzed (mean age, 44.4 years; female, 68.9%), of whom 54.8% and 45.2% were enrolled by primary care clinicians and specialists, respectively. Most patients were classified with mild asthma (76.0%, GINA treatment steps 1−2) and were overweight or obese (57.0%). Only 19.5% of patients reported full healthcare reimbursement, with 59% receiving no healthcare reimbursement. The mean asthma duration of patients was 13.5 years. Asthma was partly controlled/uncontrolled in 78.0% of patients, with 61.5% experiencing ≥ 1 severe exacerbation in the preceding 12 months. Crucially, 71.9% of patients were prescribed ≥ 3 SABA canisters, defined as over-prescription; 34.8% were prescribed ≥ 10 SABA canisters. Additionally, 38.8% of patients purchased SABA OTC, of whom 66.2% purchased ≥ 3 SABA canisters. Among patients with both SABA purchases and prescriptions, 95.5% and 57.1% had prescriptions for ≥ 3 and ≥ 10 SABA canisters, respectively. Inhaled corticosteroids (ICS), ICS with a long-acting β_2_-agonist fixed-dose combination, and oral corticosteroid bursts were prescribed to 58.8%, 24.7%, and 22.7% of patients, respectively.

**Conclusions:**

SABA over-prescription occurred in almost three-quarters of patients, with over one-third of patients purchasing SABA OTC. Therefore, SABA over-prescription is a major public health concern in Kenya, underscoring an urgent need to align clinical practices with latest evidence-based recommendations.

**Supplementary Information:**

The online version contains supplementary material available at 10.1186/s12875-023-02030-8.

## Background

Asthma, one of the most common chronic respiratory diseases, is estimated to affect 339 million people worldwide [1, 2], with current trends suggesting that an additional 100 million people may have asthma by 2025 [3]. Notably, the prevalence of asthma has increased across Africa over the past two decades [4], primarily due to rapid urbanization and increased exposure to environmental and lifestyle factors [5, 6], and stood at over 119 million across the continent in 2010 [4]. While the epidemiology of asthma in Kenya has not been comprehensively described to date, it is estimated that approximately 10% of the Kenyan population, nearly 4 million people, have asthma [7], with a higher prevalence in urban than in rural areas [8].

In Kenya, as in many parts of Africa, fragile healthcare systems overburdened by infectious diseases, a lack of trained staff and diagnostic apparatus, and the absence of public-supported asthma care programs have contributed to the high burden of asthma [7, 9, 10]. Despite improvements in healthcare delivery, the availability and affordability of drugs for the management of asthma remains a significant barrier to optimal care in Kenya [11–14], with 82% of women and 79% of men lacking health insurance coverage [15]. High rates of out-of-pocket expenditure for outpatient services, accounting for approximately 78% of the total household expenditure in Kenya [16], have further reduced the affordability of essential asthma medications, such as inhaled corticosteroids (ICS). Moreover, easy access to short-acting β_2_-agonist (SABA) relievers, coupled with the nonavailability of ICS-containing controller medication in many African countries, including Kenya [17], may explain the low levels of asthma control reported across Africa [18–21]. Notably, SABA overuse is globally associated with an increased risk of exacerbations, hospitalizations, and even mortality [22–25]. Consequently, following a landmark update in 2019 [26], the Global Initiative for Asthma (GINA) no longer recommends SABA monotherapy, and instead now recommends low-dose ICS-formoterol as the preferred, as-needed reliever for adults and adolescents at GINA treatment steps 1 and 2, and for patients prescribed ICS-formoterol maintenance therapy at GINA treatment steps 3–5 [27]. However, efforts to update the Guidelines for Asthma Management in Kenya [7], which were developed in 2011 based on regularly updated international guidelines and recommendations, such as GINA, have been lacking over the past decade, with an update not due to be published until the end of 2022.

An understanding of how access to medication and its use impacts asthma outcomes is of vital importance, particularly in Kenya, where improving the availability and affordability of all asthma medications represents an unmet need [28]. Furthermore, an assessment of asthma medication trends, particularly SABA prescription patterns, will bring clinicians and healthcare policymakers to a better understanding of the extent of SABA overuse in Kenya, and thus ensure that treatment practices align with the latest evidence-based treatment recommendations. Therefore, the SABA use IN Asthma (SABINA) program [29] was undertaken to describe SABA prescription patterns through a series of real-world observational studies that applied a harmonized approach to data collection, evaluation, and interpretation. The SABINA III study was conducted across 23 countries in Asia–Pacific, Africa, the Middle East, Latin America, and in Russia [30]. Here, we present the results from the Kenyan cohort of the SABINA III study to provide real-world evidence on asthma treatment practices in this country.

## Methods

### Study design

The SABINA III study methodology has been published previously [30]. In brief, this was an observational, cross-sectional study conducted at 19 sites across Kenya, with patient recruitment from August 1, 2019, to November 30, 2019. The objectives of this study were to describe the demographic and clinical features of the asthma population by asthma severity, and to estimate the number of SABA (canisters per year) and ICS (by average daily dose: low, medium, or high) prescriptions per patient and within the different SABA and ICS groups. Prespecified patient data on exacerbation history, comorbidities, and asthma medication prescriptions were collected from existing medical records by healthcare providers (HCPs) and collated into electronic case report forms (eCRFs) during a single study visit at each site. The study was conducted in compliance with the study protocol and the Declaration of Helsinki, with approval received from the African Medical and Research Foundation (AMREF) Ethical and Scientific Review Committee (approval number P618/2019).

### Study population

Patients aged  ≥ 12 years with a physician documented diagnosis of asthma,  ≥ 3 prior consultations with their HCP, and medical records containing data for  ≥ 12 months prior to the study visit were eligible for enrollment in the study. Patients with a diagnosis of other chronic respiratory diseases, such as chronic obstructive pulmonary disease, were excluded. Signed informed consent was collected from patients or their legal guardians. Primary and specialist care potential study sites were selected using purposive sampling with the aim of obtaining a sample representative of asthma management in Kenya by a national coordinator, who also facilitated the selection of investigators.

### Study variables and outcomes

Each patient was categorized based on their SABA and ICS prescriptions in the 12 months prior to the study visit. SABA prescriptions were categorized as 0, 1–2, 3–5, 6–9, 10–12, and  ≥ 13 canisters, with the prescription of ≥ 3 SABA canisters per year defined as over-prescription [22, 24, 31]. ICS canister prescriptions in the preceding 12 months were recorded and categorized according to the prescribed average daily dose (low, medium, or high) [32].

Secondary variables included sociodemographic characteristics (number of comorbid conditions, age, gender, body mass index [BMI], smoking status, educational level [primary school, secondary school, high school, or university and/or postgraduate education], and medication reimbursement status [not reimbursed, partly reimbursed, or fully reimbursed]), practice type (primary or specialist care), asthma characteristics, investigator-classified asthma severity (guided by GINA 2017 treatment steps: steps 1–2, mild asthma; steps 3–5, moderate-to-severe asthma) [32], and time since asthma diagnosis.

Prescriptions for asthma medications in the preceding 12 months, including ICS, fixed-dose combinations of ICS with a long-acting β_2_-agonist (LABA), long-term oral corticosteroid (OCS) treatment (any OCS treatment for  > 10 days), OCS burst treatment (short course of intravenous corticosteroids or OCS administered for 3–10 days, or a single dose of an intramuscular corticosteroid to treat an exacerbation), and antibiotics for asthma, were also recorded. Data for pharmacy purchases of over-the-counter (OTC) SABA without a prescription in the previous 12 months was based on patient recall and obtained directly from patients at the study visit, which was subsequently entered in the eCRF by the investigator.

Asthma symptom control was evaluated at the time of the study visit using the GINA 2017 assessment for asthma control and categorized as well controlled, partly controlled, or uncontrolled [32]. The number of severe exacerbations in the 12 months before the study visit was based on the American Thoracic Society/European Respiratory Society recommendations and defined as a worsening of asthma symptoms resulting in hospitalization, an emergency room visit, a prescription of intravenous corticosteroids or OCS for  ≥ 3 days, or a single dose of an intramuscular corticosteroid [33].

### Statistical analysis

As previously described [30], descriptive analyses were used to characterize patients according to baseline demographics and clinical characteristics. Continuous variables were summarized by the number of nonmissing values, mean (standard deviation [SD]), and median (range). Categorical variables were summarized by frequency counts and percentages. To ensure that the overall SABINA III study was adequately powered, the aim was to enroll up to 500 patients from each participating country, with 20–25 patients recruited from each participating site.

## Results

### Study population

**Fig. 1:**
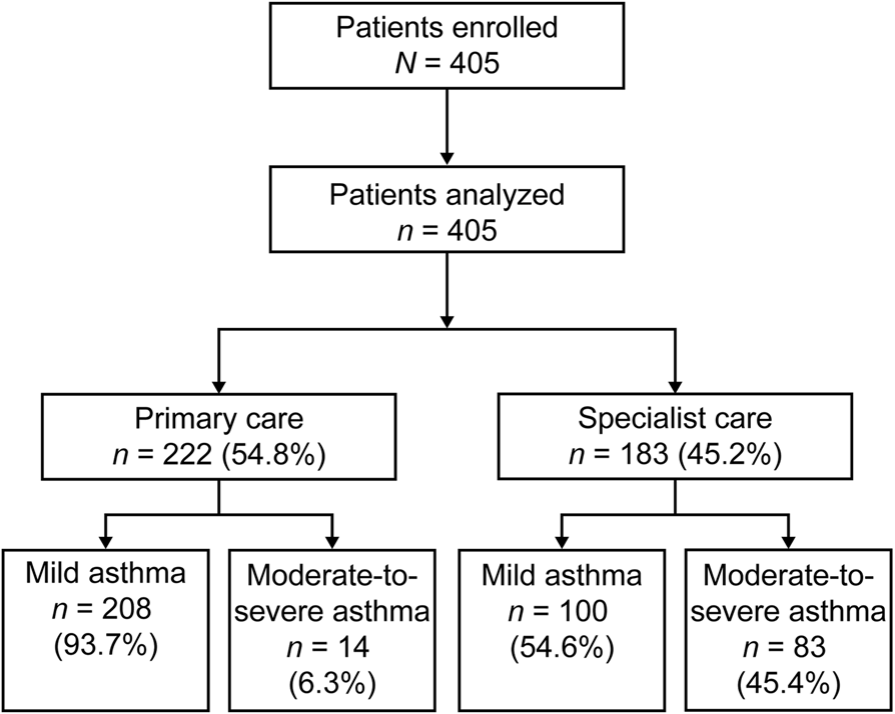
Patient disposition and study population by practice type and investigator-classified asthma severity

Overall, 405 patients were enrolled in the study, all of whom were included in the analysis. A slightly higher proportion of patients were treated by primary care clinicians than by specialists (54.8% and 45.2%, respectively; Fig. 1).

### Patient characteristics

Overall, the mean (SD) age of patients was 44.4 (14.0) years, with more than three-quarters of patients (76.3%) aged 18–54 years (Table 1). The majority of patients were female (68.9%) and had never smoked (87.9%). The mean (SD) BMI of patients was 26.2 (5.1) kg/m^2^, with 57.0% being overweight or obese (BMI  ≥ 25 kg/m^2^). More than one-third of patients (40.2%) had received secondary or high school education, whereas 20% had obtained university and/or postgraduate education. A higher proportion of patients treated in specialist care had university and/or postgraduate education compared with those treated in primary care (32.2% vs 9.9%). Overall, only 19.5% of patients reported full healthcare reimbursement, with 59% receiving no healthcare reimbursement. Notably, a higher proportion of patients under specialist care reported full healthcare reimbursement compared with those under primary care (33.9% vs 7.7%); additionally, in specialist care, more patients with moderate-to-severe asthma reported full healthcare reimbursement compared with those with mild asthma (48.2% vs 22%). Approximately three-quarters of patients (74.1%) had no comorbidities, with 24.9% reporting 1–2 comorbidities.

**Table 1:** Sociodemographic and asthma characteristics according to investigator-classified asthma severity and practice type

Sociodemographic and asthma characteristics	All (*N* = 405)		Primary care (*n* = 222)				Specialist care (*n* = 183)		
			**Mild asthma (*n* = 208)**	**Moderate-to-severe asthma (*n* = 14)**	**All (*n* = 222)**		**Mild asthma (*n* = 100)**	**Moderate-to-severe asthma** **(*n* = 83)**	**All (*n* = 183)**
**Sociodemographic characteristics**									
**Age (years), mean (SD)**	44.4 (14.0)		44.1 (13.6)	48.0 (9.5)	44.3 (13.4)		44.4 (14.5)	44.6 (14.9)	44.5 (14.7)
**Age groups**									
12–17 years	0 (0)		0 (0)	0 (0)	0 (0)		0 (0)	0 (0)	0 (0)
≥ 18–54 years	309 (76.3)		163 (78.4)	12 (85.7)	175 (78.8)		72 (72)	62 (74.7)	134 (73.2)
≥ 55 years	96 (23.7)		45 (21.6)	2 (14.3)	47 (21.2)		28 (28)	21 (25.3)	49 (26.8)
Total	405		208	14	222		100	83	183
**Sex (female)**	279 (68.9)		146 (70.2)	10 (71.4)	156 (70.3)		66 (66)	57 (68.7)	123 (67.2)
**BMI (kg/m**^**2**^**)**									
Mean (SD)	26.2 (5.1)		26.2 (5.1)	27.3 (7.1)	26.3 (5.3)		26.4 (5.0)	25.8 (4.6)	26.1 (4.8)
Median (min, max)	25.9 (16.3, 42.5)		25.9 (16.3, 42.5)	25.6 (18.0, 40.9)	25.9 (16.3, 42.5)		26.2 (16.9, 42.5)	25.7 (16.3, 41.8)	26.0 (16.3, 42.5)
**BMI group (kg/m**^**2**^**)**									
< 18.5	22 (5.4)		9 (4.3)	1 (7.1)	10 (4.5)		6 (6)	6 (7.2)	12 (6.6)
≥ 18.5–24.9	152 (37.5)		83 (39.9)	6 (42.9)	89 (40.1)		34 (34)	29 (34.9)	63 (34.4)
≥ 25–29.9	150 (37)		72 (34.6)	2 (14.3)	74 (33.3)		42 (42)	34 (41)	76 (41.5)
≥ 30	81 (20)		44 (21.2)	5 (35.7)	49 (22.1)		18 (18)	14 (16.9)	32 (17.5)
**Smoking status history**									
Active smoker	4 (1)		3 (1.4)	1 (7.1)	4 (1.8)		0 (0)	0 (0)	0 (0)
Former smoker	45 (11.1)		24 (11.5)	2 (14.3)	26 (11.7)		12 (12)	7 (8.4)	19 (10.4)
Nonsmoker	356 (87.9)		181 (87)	11 (78.6)	192 (86.5)		88 (88)	76 (91.6)	164 (89.6)
**Number of comorbidities**									
0	300 (74.1)		149 (71.6)	11 (78.6)	160 (72.1)		82 (82)	58 (69.9)	140 (76.5)
1–2	101 (24.9)		57 (27.4)	3 (21.4)	60 (27)		18 (18)	23 (27.7)	41 (22.4)
3–4	3 (0.7)		2 (1)	0 (0)	2 (0.9)		0 (0)	1 (1.2)	1 (0.5)
≥ 5	1 (0.2)		0 (0)	0 (0)	0 (0)		0 (0)	1 (1.2)	1 (0.5)
**Education level**									
Primary school	154 (38)	99 (47.6)		4 (28.6)	103 (46.4)	26 (26)		25 (30.1)	51 (27.9)
Secondary school	84 (20.7)	52 (25)		1 (7.1)	53 (23.9)	19 (19)		12 (14.5)	31 (16.9)
High school	79 (19.5)	35 (16.8)		4 (28.6)	39 (17.6)	27 (27)		13 (15.7)	40 (21.9)
University and/or postgraduate education	81 (20)	17 (8.2)		5 (35.7)	22 (9.9)	26 (26)		33 (39.8)	59 (32.2)
Unknown	7 (1.7)	5 (2.4)		0 (0)	5 (2.3)	2 (2)		0 (0)	2 (1.1)
**Healthcare insurance/medication funding**									
Not reimbursed	239 (59)	131 (63)		8 (57.1)	139 (62.6)	63 (63)		37 (44.6)	100 (54.6)
Partially reimbursed	83 (20.5)	66 (31.7)		0 (0)	66 (29.7)	11 (11)		6 (7.2)	17 (9.3)
Fully reimbursed	79 (19.5)	11 (5.3)		6 (42.9)	17 (7.7)	22 (22)		40 (48.2)	62 (33.9)
Unknown	4 (1)	0 (0)		0 (0)	0 (0)	4 (4)		0 (0)	4 (2.2)
**Asthma characteristics**									
**Asthma duration (years)**									
Mean (SD)	13.5 (11.4)		13.9 (11.2)	22.2 (14.6)	14.4 (11.6)		13.0 (11.8)	11.7 (10.0)	12.4 (11.0)
Median (min, max)	10.0 (1.0, 58.0)		11.0 (1.0, 58.0)	19.5 (4.0, 52.0)	11.0 (1.0, 58.0)		9.5 (1.0, 50.0)	8.0 (1.0, 47.0)	9.0 (1.0 50.0)
**Number of severe asthma exacerbations 12 months before the study visit**									
Mean (SD)	2.1 (3.8)		2.3 (4.8)	1.6 (1.3)	2.2 (4.6)		2.0 (1.7)	2.0 (3.3)	2.0 (2.6)
Median (min, max)	1.0 (0.0, 36.0)		1.0 (0.0, 36.0)	1.5 (0.0, 4.0)	1.0 (0.0, 36.0)		2.0 (0.0, 10.0)	1.0 (0.0, 20.0)	1.0 (0.0, 20.0)
**Number of severe asthma exacerbations 12 months before the study visit**									
0	156 (38.5)		89 (42.8)	4 (28.6)	93 (41.9)		25 (25)	38 (45.8)	63 (34.4)
1	71 (17.5)		39 (18.8)	3 (21.4)	42 (18.9)		16 (16)	13 (15.7)	29 (15.8)
2	66 (16.3)		29 (13.9)	3 (21.4)	32 (14.4)		24 (24)	10 (12)	34 (18.6)
≥ 3	112 (27.7)		51 (24.5)	4 (28.6)	55 (24.8)		35 (35)	22 (26.5)	57 (31.1)
**GINA classification**									
Step 1	71 (17.5)		41 (19.7)	0 (0)	41 (18.5)		30 (30)	0 (0)	30 (16.4)
Step 2	237 (58.5)		167 (80.3)	0 (0)	167 (75.2)		70 (70)	0 (0)	70 (38.3)
Step 3	70 (17.3)		0 (0)	8 (57.1)	8 (3.6)		0 (0)	62 (74.7)	62 (33.9)
Step 4	27 (6.7)		0 (0)	6 (42.9)	6 (2.7)		0 (0)	21 (25.3)	21 (11.5)
Step 5	0 (0)		0 (0)	0 (0)	0 (0)		0 (0)	0 (0)	0 (0)
**Level of asthma symptom control**									
Well controlled	89 (22)		44 (21.2)	4 (28.6)	48 (21.6)		11 (11)	30 (36.1)	41 (22.4)
Partly controlled	187 (46.2)		88 (42.3)	5 (35.7)	93 (41.9)		62 (62)	32 (38.6)	94 (51.4)
Uncontrolled	129 (31.9)		76 (36.5)	5 (35.7)	81 (36.5)		27 (27)	21 (25.3)	48 (26.2)

Data are presented as *n* (%) unless otherwise specified

*BMI* Body mass index, *GINA* Global Initiative for Asthma, *max* Maximum, *min* Minimum, *SD* Standard deviation

### Disease characteristics

Patients had a mean (SD) asthma duration of 13.5 (11.4) years (Table 1). Overall, 76.0% of patients were classified with mild asthma (GINA treatment steps 1–2) and 24.0% with moderate-to-severe asthma (GINA treatment steps 3–5); most patients were at GINA treatment step 2 (58.5%). Patients reported a mean (SD) of 2.1 (3.8) severe asthma exacerbations, with 61.5% of patients experiencing  ≥ 1 severe asthma exacerbation in the 12 months preceding study initiation. A slightly higher proportion of patients under specialist care reported  ≥ 1 severe asthma exacerbation compared with those under primary care (65.6% vs 58.1%), with this occurring in more patients with mild than with moderate-to-severe asthma under specialist care (75.0% vs 54.2%). The level of asthma symptom control was assessed as well controlled in 22.0%, partly controlled in 46.2%, and uncontrolled in 31.9% of patients. Although the percentage of patients with well-controlled asthma was comparable across primary and specialist care (21.6% and 22.4%, respectively), a higher proportion of patients in primary care had uncontrolled asthma compared with those in specialist care (36.5% vs 26.2%).

### Asthma treatments in the 12 months before the study visit

#### SABA prescription categorization

**Fig. 2:**
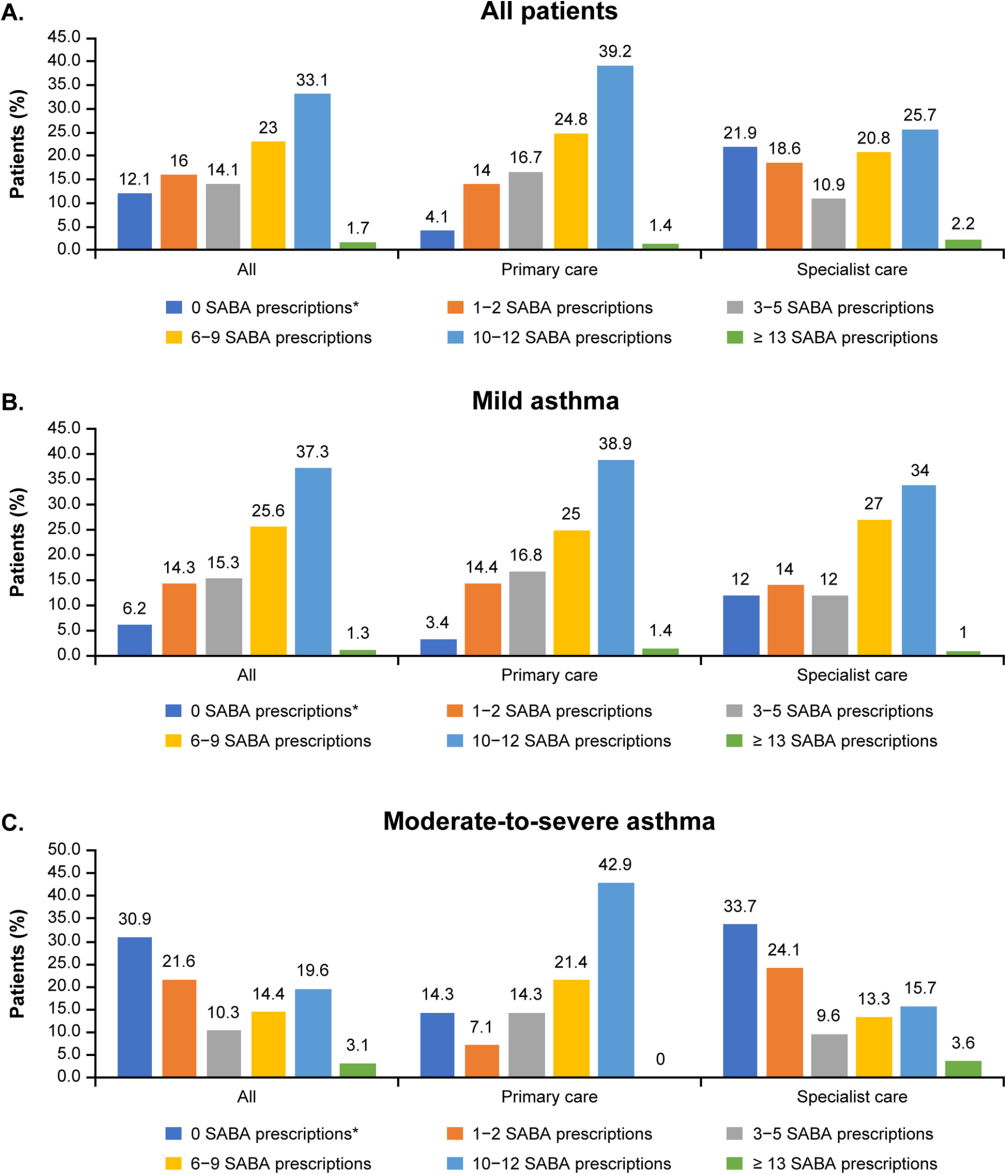
Proportion of patients receiving SABA prescriptions in the 12 months before the study visit. Patients were categorized according to investigator-classified asthma severity and practice type. **A** All patients, **B** Mild asthma, **C** Moderate-to-severe asthma. *Patients without SABA prescriptions did not report what reliever they were using. SABA, Short-acting β_2_-agonist

Overall, 71.9% of patients were prescribed  ≥ 3 SABA canisters, defined as over-prescription, in the 12 months prior to the study. Moreover, 34.8% of patients were prescribed  ≥ 10 SABA canisters. Altogether, 12.1% of patients received no SABA prescriptions (Fig. 2). More patients with mild asthma than with moderate-to-severe asthma were prescribed  ≥ 3 (79.5% vs 47.4%) and  ≥ 10 (38.6% vs 22.7%) SABA canisters in the previous 12 months. A higher proportion of patients with mild asthma treated in primary care compared with specialist care were prescribed  ≥ 3 (82.2% vs 74%) and  ≥ 10 (40.4% vs 35%) SABA canisters in the preceding 12 months. Similarly, more patients with moderate-to-severe asthma treated in primary care versus specialist care were prescribed ≥ 3 (78.6% vs 42.2%) and  ≥ 10 (42.9% vs 19.3%) canisters in the 12 months prior.

#### SABA monotherapy

Overall, 15.3% of patients, all of whom were categorized with mild asthma, were prescribed SABA monotherapy, with a mean (SD) of 7.6 (4.0) SABA canisters in the previous 12 months; of these patients, 85.5% were prescribed  ≥ 3 canisters and 43.5%  ≥ 10 canisters (Table 2). More patients under specialist care were prescribed  ≥ 3 SABA canisters compared with those under primary care (95.7% vs 79.5%). In contrast, a higher proportion of patients in primary care were prescribed  ≥ 10 SABA canisters compared with those in specialist care (53.8% vs 26.1%).

**Table 2:** SABA prescriptions in the 12 months before the study visit

SABA prescriptions 12 months before the study visit	All (*N* = 405)	Primary care (*n* = 222)			Specialist care (*n* = 183)		
		**Mild asthma (*n* = 208)**	**Moderate-to-severe asthma (*n* = 14)**	**All** **(*n* = 222)**	**Mild asthma (*n* = 100)**	**Moderate-to-severe asthma (*n* = 83)**	**All** **(*n* = 183)**
**Patients prescribed SABA monotherapy**							
Yes	62 (15.3)	39 (18.8)	0 (0)	39 (17.6)	23 (23)	0 (0)	23 (12.6)
No	343 (84.7)	169 (81.2)	14 (100)	183 (82.4)	77 (77)	83 (100)	160 (87.4)
*Number of canisters/inhalers prescribed per patient 12 months before the study visit*							
Mean (SD)	7.6 (4.0)	7.9 (4.3)	NA	7.9 (4.3)	6.9 (3.2)	NA	6.9 (3.2)
Median (min, max)	6.0 (1.0, 14.0)	10.0 (1.0, 14.0)	NA	10.0 (1.0, 14.0)	6.0 (2.0, 12.0)	NA	6.0 (2.0, 12.0)
*Number of prescriptions 12 months before the study visit (canisters/inhalers) by category*							
1–2	9 (14.5)	8 (20.5)	NA	8 (20.5)	1 (4.3)	NA	1 (4.3)
3–5	10 (16.1)	5 (12.8)	NA	5 (12.8)	5 (21.7)	NA	5 (21.7)
6–9	16 (25.8)	5 (12.8)	NA	5 (12.8)	11 (47.8)	NA	11 (47.8)
10–12	26 (41.9)	20 (51.3)	NA	20 (51.3)	6 (26.1)	NA	6 (26.1)
≥ 13	1 (1.6)	1 (2.6)	NA	1 (2.6)	0 (0)	NA	0 (0)
Total	62	39	NA	39	23	NA	23
**Patients prescribed SABA in addition to maintenance therapy**							
Yes	294 (72.6)	162 (77.9)	12 (85.7)	174 (78.4)	65 (65)	55 (66.3)	120 (65.6)
No	111 (27.4)	46 (22.1)	2 (14.3)	48 (21.6)	35 (35)	28 (33.7)	63 (34.4)
*Number of canisters/inhalers prescribed per patient 12 months before the study visit*							
Mean (SD)	7.3 (4.3)	7.5 (3.9)	8.0 (3.8)	7.6 (3.9)	7.6 (4.1)	6.1 (5.5)	6.9 (4.9)
Median (min, max)	6.0 (1.0, 30.0)	6.0 (1.0, 14.0)	8.0 (1.0, 12.0)	6.0 (1.0, 14.0)	7.0 (1.0, 14.0)	4.0 (1.0, 30.0)	6.0 (1.0, 30.0)
*Number of prescriptions 12 months before the study visit (canisters/inhalers) by category*							
1–2	56 (19)	22 (13.6)	1 (8.3)	23 (13.2)	13 (20)	20 (36.4)	33 (27.5)
3–5	47 (16)	30 (18.5)	2 (16.7)	32 (18.4)	7 (10.8)	8 (14.5)	15 (12.5)
6–9	77 (26.2)	47 (29)	3 (25)	50 (28.7)	16 (24.6)	11 (20)	27 (22.5)
10–12	108 (36.7)	61 (37.7)	6 (50)	67 (38.5)	28 (43.1)	13 (23.6)	41 (34.2)
≥ 13	6 (2)	2 (1.2)	0 (0)	2 (1.1)	1 (1.5)	3 (5.5)	4 (3.3)
Total	294	162	12	174	65	55	120

All data are described as *n* (%) unless otherwise specified

*max* Maximum, *min* Minimum, *NA* Not applicable, *SABA* Short-acting β_2_-agonist, *SD* Standard deviation

#### SABA in addition to maintenance therapy

The majority of patients (72.6%) were prescribed SABA in addition to maintenance therapy, with a mean (SD) of 7.3 (4.3) canisters in the preceding 12 months. Among these patients, 81.0% and 38.8% were prescribed  ≥ 3 and  ≥ 10 SABA canisters, respectively (Table 2). A higher proportion of patients in primary care were prescribed  ≥ 3 SABA canisters compared with those under specialist care (86.8% vs 72.5%), whereas a comparable proportion of patients in primary and specialist care were prescribed  ≥ 10 SABA canisters in the previous 12 months (39.7% and 37.5%, respectively).

#### SABA purchase without a prescription

**Fig. 3:**
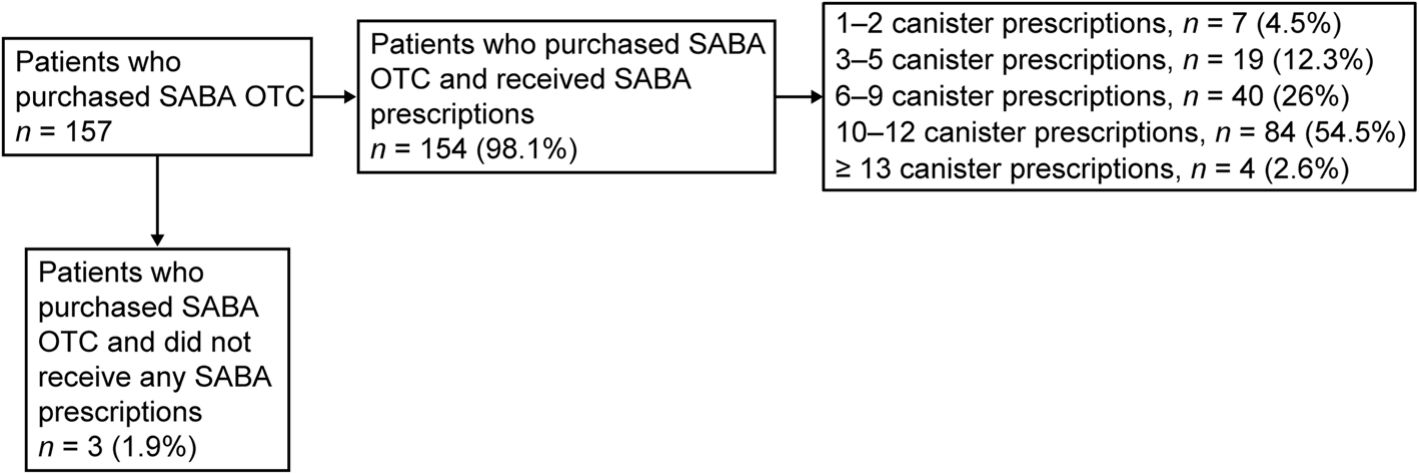
SABA purchases and prescriptions in patients with asthma. OTC, Over-the-counter; SABA, Short-acting β_2_-agonist

Overall, 38.8% of patients purchased SABA OTC, of whom 66.2% purchased  ≥ 3 SABA canisters in the 12 months prior to study entry (Table 3). Almost all patients (98.1%) who purchased SABA OTC had also received SABA prescriptions. Among patients with both SABA purchases and prescriptions, 95.5% had prescriptions for  ≥ 3 SABA canisters and 57.1% had prescriptions for ≥ 10 SABA canisters in the previous 12 months (Fig. 3). Patients treated by specialists had slightly more SABA purchases than those treated by primary care clinicians (42.1% vs 36%; Table 3).

**Table 3:** SABA OTC purchase in the 12 months before the study visit

SABA OTC 12 months before the study visit	All (*N* = 405)	Primary care (*n* = 222)			Specialist care (*n* = 183)		
		**Mild asthma (*n* = 208)**	**Moderate-to-severe asthma (*n* = 14)**	**All** **(*n* = 222)**	**Mild asthma (*n* = 100)**	**Moderate-to-severe asthma (*n* = 83)**	**All** **(*n* = 183)**
**Additional SABA without a prescription from the pharmacy 12 months before the study visit**							
Yes	157 (38.8)	75 (36.1)	5 (35.7)	80 (36)	55 (55)	22 (26.5)	77 (42.1)
No	248 (61.2)	133 (63.9)	9 (64.3)	142 (64)	45 (45)	61 (73.5)	106 (57.9)
Total	405	208	14	222	100	83	183
**Number of additional SABA 12 months before the study visit (canisters)**							
1–2	53 (33.8)	33 (44)	1 (20)	34 (42.5)	16 (29.1)	3 (13.6)	19 (24.7)
3–5	77 (49)	32 (42.7)	3 (60)	35 (43.8)	29 (52.7)	13 (59.1)	42 (54.5)
6–9	21 (13.4)	5 (6.7)	1 (20)	6 (7.5)	9 (16.4)	6 (27.3)	15 (19.5)
10–12	6 (3.8)	5 (6.7)	0 (0)	5 (6.2)	1 (1.8)	0 (0)	1 (1.3)
≥ 13	0 (0)	0 (0)	0 (0)	0 (0)	0 (0)	0 (0)	0 (0)
Total	157	75	5	80	55	22	77

Data are presented as *n* (%) unless otherwise specified

*OTC* Over-the-counter, *SABA* Short-acting β_2_-agonist

### Prescriptions for other asthma treatments

#### Inhaled corticosteroids

Overall, 58.8% of patients were prescribed ICS maintenance therapy, with a mean (SD) of 9.6 (3.7) ICS canisters in the preceding 12 months (Supplementary Table 1). Most patients were prescribed medium-dose ICS (76.8%), whereas 14.3% and 8.9% of patients were prescribed low- and high-dose ICS, respectively. Nearly three-quarters (74.3%) of patients were prescribed ICS in primary care compared with only 39.9% in specialist care.

#### ICS/LABA fixed-dose combination

Overall, 24.7% of patients, the majority of whom had moderate-to-severe asthma, were prescribed an ICS/LABA fixed-dose combination as maintenance therapy, with 80.0% receiving medium-dose ICS. Compared with 47% of patients in specialist care who were prescribed an ICS/LABA fixed-dose combination, only 6.3% of patients in primary care were prescribed this medication (Supplementary Table 1).

#### Other asthma medications

Overall, in the 12 months prior to study entry, 22.7% of patients were prescribed an OCS burst, with prescription rates comparable in patients across primary and specialist care (23.9% and 21.3%, respectively; Supplementary Table 1). In addition, 14.1% of patients were prescribed an antibiotic (13.1% in primary care and 15.4% in specialist care; Supplementary Table 1).

## Discussion

Results from the Kenyan cohort of the SABINA III study provide valuable real-world evidence on asthma management practices in this country, which until now has received relatively little attention. Notably, 71.9% of patients overall were prescribed SABA in excess of current treatment recommendations (≥ 3 SABA canisters/year), which translated into a high disease burden, emphasizing an urgent need for improvements in asthma care.

In general, the overall sociodemographic and disease characteristics of patients from Kenya were consistent with those in SABINA III [30], although a few notable differences were observed. In Kenya, 54.8% of patients were treated in primary care, which was considerably higher than that observed in SABINA III (17.2%). Consequently, a higher proportion of patients in SABINA Kenya had mild asthma compared with those in SABINA III (76.5% vs 23.4%, respectively) [30]. Strikingly, only 19.5% of patients in Kenya reported fully reimbursed healthcare compared with 47.2% of patients in SABINA III [30]. Interestingly, only 7.7% of patients in primary care reported full healthcare reimbursement compared with 33.9% in specialist care. This finding may be attributable to the fact that patients under specialist care are more likely to have private healthcare insurance. Moreover, in specialist care, 48.2% of patients with moderate-to-severe asthma were fully reimbursed for healthcare compared with only 22% of patients with mild asthma. This could be explained by the observation that patients with moderate-to-severe asthma are more likely to claim their healthcare insurance than those with mild asthma due to rising healthcare costs associated with increasing asthma severity [14, 22]. However, the high percentage of patients with mild asthma who reported  ≥ 1 severe exacerbation in the previous 12 months in this study underscores the need for patients to reconsider how they utilize their healthcare insurance to ensure optimal treatment.

Overall, a high proportion of patients in Kenya were prescribed SABA treatments. Although only 15.3% of patients were prescribed SABA monotherapy, 85.5% of these were prescribed  ≥ 3 SABA canisters in the preceding 12 months, which is considered over-prescription. Similarly, of the 72.6% of patients who were prescribed SABA in addition to maintenance therapy, 81.0% were overprescribed SABA. Worryingly, 43.5% and 38.8% of patients were prescribed ≥ 10 canisters of SABA as monotherapy and with maintenance treatment, respectively. Therefore, taken together, nearly three-quarters (71.9%) of all patients were prescribed  ≥ 3 SABA canisters in the 12 months prior, with 34.8% prescribed  ≥ 10 SABA canisters. This is of concern as aggregated SABINA III data from 24 countries suggested an association between high SABA prescriptions and poor clinical outcomes, with prescriptions of  ≥ 3 SABA canisters (vs 1 − 2) being associated with increasingly lower odds of controlled or partly controlled asthma, and higher rates of severe exacerbations [30]. Although SABA over-prescription occurred in both primary and specialist care, this trend was more apparent in primary care, likely reflecting the inherent challenges faced by primary care clinicians, including limited consultation times and a lack of diagnostic resources [34–36]. Other potential explanations for this observation are the fact that most asthma guidelines are generally biased toward a secondary care perspective, thereby limiting their implementation in a primary care setting; unfamiliarity of primary care clinicians with GINA recommendations [35]; and a time lag between revisions to GINA and subsequent updates of local guidelines. Notably, SABA over-prescription was more common in patients with mild asthma; in line with previous reports in the literature, this may be due to the potential underestimation of patients with milder disease [37–39], resulting in inappropriate management of patients with mild asthma, leading to poor symptom control. However, discrepancies between clinical and objective assessments of asthma may also have led to a misclassification of asthma severity [40], resulting in a proportion of patients with moderate-to-severe asthma not being adequately captured.

Notably, not all SABAs were obtained with prescriptions; over one-third of patients (38.8%) from Kenya purchased SABA OTC, of whom 66.2% purchased  ≥ 3 canisters. Alarmingly, in nearly all cases (98.1%), these SABA canisters were purchased in addition to those prescribed by clinicians, with 95.5% and 57.1% of these patients already receiving prescriptions for  ≥ 3 SABA and  ≥ 10 SABA canisters, respectively, in the previous 12 months. Although limited literature is currently available on the use of OTC medications to treat asthma in Kenya, these findings were not entirely unexpected as the purchase of OTC drugs, particularly painkillers, antibiotics, and antimalarials, is widespread across the country [41–43]. In addition, the fact that nearly 60% of patients in this study reported no healthcare reimbursement, combined with high levels of out-of-pocket expenditure for medicines for noncommunicable diseases reported across Kenya [16, 44], likely further contributed to the high levels of SABA purchase observed in this study. However, this is a matter of concern because SABA purchase is associated with infrequent clinician consultations; low use of prescription medication, particularly ICS; and overall undertreatment of asthma [45–47]. Indeed, the Kenyan government is currently striving to outlaw the sale of OTC drugs in an attempt to encourage citizens to seek medical attention from qualified healthcare practitioners [43]. Therefore, our findings provide further impetus for reform, highlighting an urgent need to drive policy changes to regulate SABA purchase without prescriptions and provide affordable care for all patients with asthma in Kenya.

Altogether, over half of all patients (58.8%) were prescribed ICS, which was in alignment with the fact that the majority of patients (76.0%) had mild asthma; however, over three-quarters of patients (76.8%) received medium-dose ICS instead of the recommended low-dose ICS [27]. Reassuringly, patients were prescribed a mean of 9.6 ICS canisters in the preceding 12 months. On the basis that one canister per month is considered appropriate, this quantity suggests good clinical practice and may be indicative of automatic repeat prescriptions. However, it could not be conclusively determined whether patients took their medication as prescribed. In line with the fact that 24.0% of patients had moderate-to-severe asthma, 24.7% of patients were prescribed an ICS/LABA fixed-dose combination. Interestingly, 22.7% of patients were prescribed an OCS burst, presumably for the management of exacerbations. However, this was lower than anticipated, given that 61.5% of patients reported  ≥ 1 severe asthma exacerbation in the previous 12 months. While this finding may reflect ongoing concerns around the use of short courses of OCS with growing evidence now suggesting that even brief dosing periods of 3–7 days may increase the risk of adverse events, including loss of bone density, hypertension, and gastrointestinal ulcers/bleeds [48], it may also be a consequence of the substantial work that has been undertaken in Kenya to reduce the prescription of OCS bursts for exacerbations. Following this success, similar efforts are now required to tackle the over-prescription of SABAs. Despite the fact that GINA does not support the routine use of antibiotics for asthma unless there is strong evidence of lung infection [27], 14.1% of patients from Kenya were prescribed antibiotics for asthma. While this may be explained in part by a lack of familiarity with asthma guidelines, it may also reflect prescribing practices in Kenya, where considerable antibiotic prescriptions for numerous conditions have been reported [49], resulting in high rates of antimicrobial resistance [50] and culminating in recent research to evaluate optimal strategies for the development of stewardship programs [49].

Crucially, asthma control in Kenya was poor, with less than a quarter of patients having well-controlled asthma compared with 43.3% of patients in the overall SABINA III cohort [30]. Consequently, the burden of asthma in Kenya was high, with 61.5% of patients experiencing  ≥ 1 severe exacerbation in the previous 12 months. However, our findings are aligned with previous reports from Africa documenting suboptimal asthma control [21, 51–53]. Indeed, results from the Epidemiological Study on the Management of Asthma in Asthmatic Middle East Adult Population, a large-scale cross-sectional epidemiological study in 7236 patients that included three African countries (Algeria, Egypt, and Tunisia), reported that asthma was only controlled in approximately one-third of all patients [54]. While organizations such as the National Asthma Education Program [55] aim to promote the goals of asthma management, including the complete clinical control of asthma through the education of healthcare professionals, patients, and the general public across Africa [56], our study clearly demonstrates the need for similar country-wide clinician- and patient-centered awareness programs to improve asthma outcomes in Kenya.

The results of this study should be viewed in light of several limitations. SABA prescription data do not always reflect medication use and do not provide information on treatment adherence. The use of GINA 2017 guidelines (which were in place at the time this study was conducted) for classifying disease severity may have accounted for some of the observed high levels of SABA prescriptions. Furthermore, since data entry into the eCRFs relied on clinician assessments, findings may have been impacted by misinterpretation of instructions and incorrect patient classification or treatment. Patient-reported data on SABA OTC purchase may have been subject to recall and nonresponse bias [57, 58]. Additionally, only the number of comorbidities (categorized as 0, 1–2, 3–4, and  ≥ 5) were recorded in the eCRF, while data on the type and rate of comorbidities were not captured. Moreover, the impact of comorbidities and a range of other factors, such as gender, BMI, smoking status, patient education, healthcare reimbursement status, inhaler technique, and patient-physician communication, on asthma control were not examined in this study. Information on the management of asthma exacerbations and whether the correct treatment was prescribed was not collected. Finally, as the primary focus of this study was on SABA canister prescriptions, the potential overuse of oral (tablets) and nebulized dosage forms of SABA was not captured.

Despite these limitations, this study is the first to describe SABA prescription patterns in Kenya. Furthermore, the collection of these real-world data on SABA over-prescription in patients equally distributed across primary and specialist care provides a true representation of how asthma is currently being managed in Kenya. Overall, the pattern of high SABA over-prescription and OTC purchase indicates that urgent action is required to update national guidelines and drive policy change in Kenya. Crucially, our study highlights the need to align clinical practices with the latest evidence-based recommendations to improve asthma outcomes across the country.

## Conclusions

Results from the Kenyan cohort of the SABINA III study demonstrated SABA over-prescription (≥ 3 canisters in the previous 12 months) in nearly three-quarters of all patients (71.9%). Furthermore, over one-third of patients (38.8%) purchased SABA OTC without a prescription, of whom 66.2% purchased  ≥ 3 canisters of SABA. Almost all patients (98.1%) who purchased SABA OTC had also received SABA prescriptions. Overall, asthma control was low, with 61.5% of patients experiencing  ≥ 1 severe asthma exacerbation in the previous 12 months. Therefore, SABA over-prescription remains a major public health concern in Kenya, requiring urgent action from HCPs and policymakers to work together to update national guidelines, regulate SABA purchase without prescription, and ensure that clinical practices are aligned with the latest evidence-based recommendations.

## Supplementary Information

**Additional file 1: Supplementary Table 1.** Other asthma treatments prescribed in the 12 months before the study visit.

## Data Availability

Data underlying the findings described in this manuscript may be obtained in accordance with AstraZeneca’s data-sharing policy described at https://astrazenecagrouptrials.pharmacm.com/ST/Submission/Disclosure. Data for studies directly listed on Vivli can be requested through Vivli at https://www.vivli.org/. Data for studies not listed on Vivli could be requested through Vivli at https://vivli.org/members/enquiries-about-studies-not-listed-on-the-vivli-platform/. AstraZeneca Vivli member page is also available outlining further details: https://vivli.org/ourmember/astrazeneca/.

## Abbreviations

AMREF: African Medical and Research Foundation
BMI: Body mass index
eCRF: Electronic case report form
GINA: Global Initiative for Asthm
HCP: Healthcare provider
ICS: Inhaled corticosteroids
LABA: Long-acting β_2_-agonist
OCS: Oral corticosteroids
OTC: Over-the-counter
SABA: Short-acting β_2_-agonist
SABINA: SABA use IN Asthma
SD: Standard deviation
