# Supplementary material for: Over-prescription of short-acting β2-agonists remains a serious health concern in Kenya: results from the SABINA III study
Source: BMC Prim Care. 2023 Jul 8;24:141. doi: 10.1186/s12875-023-02030-8 (PMC10329295; doi:10.1186/s12875-023-02030-8)
Supplement: Supplementary file 1 — Additional file 1: Supplementary Table 1. Other asthma treatments prescribed in the 12 months before the study visit. [file 12875_2023_2030_MOESM1_ESM.docx]

**Supplementary Table 1** Other asthma treatments prescribed in the 12 months before the study visit

| **Asthma treatments 12 months before the study visit** | **All**  **(*N* = 405)** | | **Primary care (*n* = 222)** | | | | **Specialist care (*n* = 183)** | | |
| --- | --- | --- | --- | --- | --- | --- | --- | --- | --- |
|  |  | | **Mild asthma (*n* = 208)** | | **Moderate-to-severe asthma  (*n* = 14)** | **All**  **(*n* = 222)** | **Mild asthma (*n* = 100)** | **Moderate-to-severe asthma  (*n* = 83)** | **All**  **(*n* = 183)** |
| **Patients prescribed ICS** | | | | | | | | | |
| Yes | | 238 (58.8) | 162 (77.9) | 3 (21.4) | | 165 (74.3) | 64 (64) | 9 (10.8) | 73 (39.9) |
| No | | 167 (41.2) | 46 (22.1) | 11 (78.6) | | 57 (25.7) | 36 (36) | 74 (89.2) | 110 (60.1) |
| *Total prescribed daily ICS dose* | | | | | | | | | |
| Low dose | 34 (14.3) | | 20 (12.4) | | 1 (33.3) | 21 (12.8) | 10 (15.6) | 3 (33.3) | 13 (17.8) |
| Medium dose | 182 (76.8) | | 121 (75.2) | | 2 (66.7) | 123 (75) | 53 (82.8) | 6 (66.7) | 59 (80.8) |
| High dose | 21 (8.9) | | 20 (12.4) | | 0 (0) | 20 (12.2) | 1 (1.6) | 0 (0) | 1 (1.4) |
| Total | 237^a^ | | 161^a^ | | 3 | 164^a^ | 64 | 9 | 73 |
| *Total ICS prescriptions 12 months before the study visit (canisters/inhalers*) | | | | | | | | | |
| *n* | 238 | | 162 | | 3 | 165 | 64 | 9 | 73 |
| Mean (SD) | 9.6 (3.7) | | 10.0 (3.5) | | 9.7 (4.0) | 10.0 (3.5) | 8.6 (3.9) | 8.9 (3.0) | 8.7 (3.8) |
| Median (min, max) | 12.0  (1.0, 13.0) | | 12.0  (1.0, 13.0) | | 12.0  (5.0, 12.0) | 12.0 (1.0, 13.0) | 10.5  (1.0, 12.0) | 8.0  (6.0, 12.0) | 10.0  (1.0, 12.0) |
| **Patients prescribed ICS/LABA (fixed-dose combination)** | | | | | | | | | |
| Yes | 100 (24.7) | | 1 (0.5) | | 13 (92.9) | 14 (6.3) | 4 (4) | 82 (98.8) | 86 (47) |
| No | 305 (75.3) | | 207 (99.5) | | 1 (7.1) | 208 (93.7) | 96 (96) | 1 (1.2) | 97 (53) |
| *Total prescribed daily ICS dose* | | | | | | | | | |
| Low dose | 15 (15) | | 1 (100) | | 2 (15.4) | 3 (21.4) | 0 (0) | 12 (14.6) | 12 (14) |
| Medium dose | 80 (80) | | 0 (0) | | 11 (84.6) | 11 (78.6) | 4 (100) | 65 (79.3) | 69 (80.2) |
| High dose | 5 (5) | | 0 (0) | | 0 (0) | 0 (0) | 0 (0) | 5 (6.1) | 5 (5.8) |
| Total | 100 | | 1 | | 13 | 14 | 4 | 82 | 86 |
| **Patients prescribed short-course OCS** | | | | | | | | | |
| Yes | 92 (22.7) | | 50 (24) | | 3 (21.4) | 53 (23.9) | 30 (30) | 9 (10.8) | 39 (21.3) |
| No | 313 (77.3) | | 158 (76) | | 11 (78.6) | 169 (76.1) | 70 (70) | 74 (89.2) | 144 (78.7) |
| **Patients prescribed antibiotics for asthma** | | | | | | | | | |
| Yes | 57 (14.1) | | 26 (12.5) | | 3 (21.4) | 29 (13.1) | 17 (17.2) | 11 (13.3) | 28 (15.4) |
| No | 347 (85.9)^a^ | | 182 (87.5) | | 11 (78.6) | 193 (86.9) | 82 (82.8)^a^ | 72 (86.7) | 154 (84.6)^a^ |

^a^Data for one patient are missing.

Data are presented as *n* (%) unless otherwise specified.

*ICS* Inhaled corticosteroids, *LABA* Long-acting β_2_-agonist, *max* Maximum, *min* Minimum, *OCS* Oral corticosteroids, *SD* Standard deviation
